# Identification of the Transgenic Integration Site in Immunodeficient tgε26 Human CD3ε Transgenic Mice

**DOI:** 10.1371/journal.pone.0014391

**Published:** 2010-12-22

**Authors:** Izumi Ohigashi, Yuki Yamasaki, Tsukasa Hirashima, Yousuke Takahama

**Affiliations:** 1 Division of Experimental Immunology, Institute for Genomic Research, University of Tokushima, Tokushima, Japan; 2 Otsuka Pharmaceutical, Tokushima, Japan; Dana-Farber Cancer Institute, United States of America

## Abstract

A strain of human CD3ε transgenic mice, tgε26, exhibits severe immunodeficiency associated with early arrest of T cell development. Complete loss of T cells is observed in homozygous tgε26 mice, but not in heterozygotes, suggesting that genomic disruption due to transgenic integration may contribute to the arrest of T cell development. Here we report the identification of the transgenic integration site in tgε26 mice. We found that multiple copies of the human CD3ε transgene are inserted between the *Sstr5* and *Metrn* loci on chromosome 17, and that this is accompanied by duplication of the neighboring genomic region spanning 323 kb. However, none of the genes in this region were abrogated. These results suggest that the severe immunodeficiency seen in tgε26 mice is not due to gene disruption resulting from transgenic integration.

## Introduction

Multiple strains of transgenic mice carrying high copy numbers of the human CD3ε (hCD3ε) genomic sequence exhibit inhibited development of T cells and natural killer cells [1]. It has been suggested that an aberrant signal generated by the transgenic hCD3ε chain contributes to abrogated development of T cells and natural killer cells [1], [2]. However, among hCD3ε transgenic lines, the strain known as tgε26 exhibits a unique and severe T cell deficiency, in which T cell development is completely arrested at an early stage in immature thymocytes before the expression of CD4 or CD8 [1]–[3]. Defective T cell development in tgε26 mice is intrinsic to lymphoid progenitor cells rather than thymic stromal cells, and is accompanied by accumulation of B cells in the thymus [3]. Interestingly, the arrest of T cell development in tgε26 mice is specific for homozygotes and is not seen in heterozygotes [1]–[3]. Thus, it has been proposed that genomic alterations associated with transgenic integration might contribute to perturbed T cell development in tgε26 mice [3], [4]. Since tgε26 mice are widely used as a model of immunodeficiency [5]–[9], we sought to identify their transgenic integration site.

## Results and Discussion

### The tgε26 transgene is inserted between the Sstr5 and Metrn loci on chromosome 17

**Figure 1:**
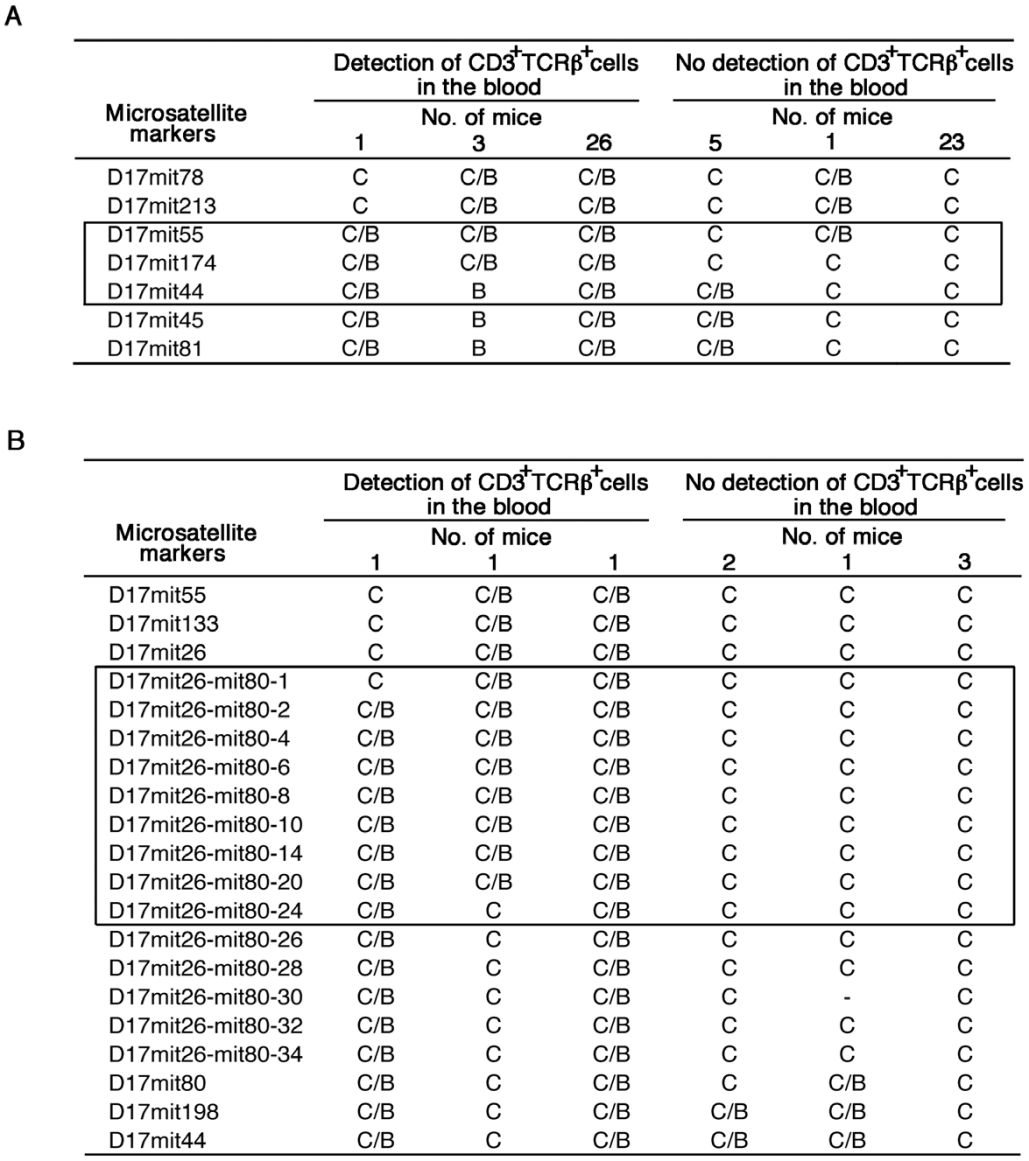
Linkage analysis of chromosome 17 with T cell deficiency in tgε26 mice. Homozygous tgε26 mice were crossed with tgε26×C57BL/6 heterozygous mice. Peripheral blood cells from the offspring were 2-color stained for CD3 and TCRβ. (A) Genomic DNA from 30 CD3^+^TCRβ^+^ cell-positive mice and 29 CD3^+^TCRβ^+^ cell-negative mice was assessed for the indicated microsatellite markers. (B) Genomic DNA from 3 CD3^+^TCRβ^+^ cell-positive mice and 6 CD3^+^TCRβ^+^ cell-negative mice was assessed for the indicated microsatellite markers. C: CBA/J homozygous alleles, C/B: CBA/J and C57BL/6 heterozygous alleles, B: C57BL/6 homozygous alleles, -: not detected. Sequences of microsatellite markers located between D17mit26 and D17mit80 are listed in Supplementary Table S1.

To determine the location of the transgene integration site in tgε26 mice, we first performed fluorescence *in situ* hybridization analysis of tgε26^+/+^ cells using the hCD3ε transgenic construct as a probe. A single signal was detected in the A2-B region of chromosome 17 (data not shown). We then analyzed the linkage between T cell deficiency and microsatellite markers on chromosome 17. This transgenic line was originally generated in (C57BL/6×CBA/J) F_2_ mice and maintained by backcrossing with C57BL/6 mice [1]. We found that loss of CD3^+^TCRβ^+^ T cells in the blood was associated with homozygosity for CBA/J-derived microsatellite markers on chromosome17. We determined that the linkage between T cell deficiency and CBA/J-derived microsatellite markers mapped to the region between D17mit55 and D17mit44 (Fig. 1A), and further narrowed down the region to the area between D17mit26-mit80-1 and D17mit 26-mit80-24, which spans approximately 1 Mb (Fig. 1B).

**Figure 2:**
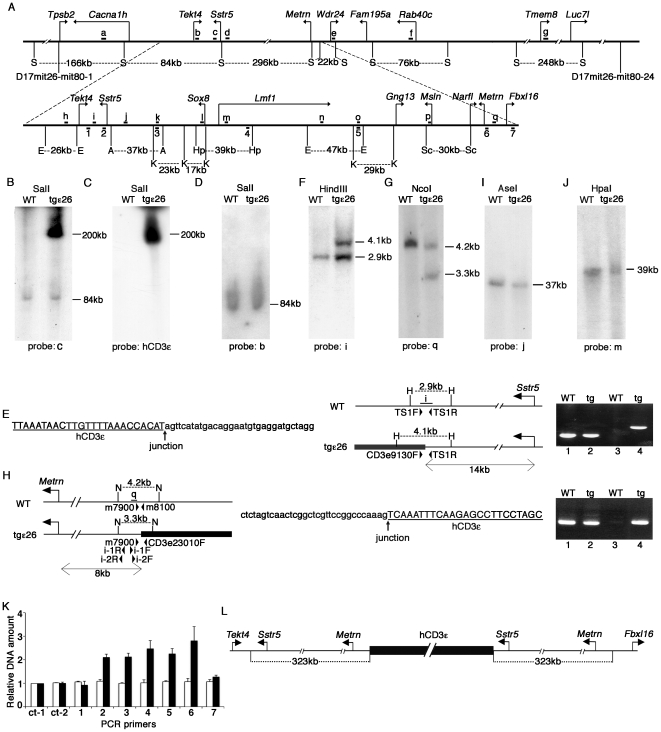
Identification of the tgε26 transgenic integration site. (A) Physical map of the genomic region between D17mit26-mit80-1 and D17mit 26-mit80-24. a–q: probes for Southern blot analysis. Details of these probes are listed in Supplementary 2. 1–7: regions of quantitative genomic PCR analysis. S: SalI, E: EcoRI, A: AseI, K: KpnI, Hp: HpaI, Sc: SacI. (B–D) PFGE Southern blot analysis of SalI-digested WT and tgε26^+/+^ genomic DNA using the indicated probes. (E) Sequence of *Sstr5*-sequence-containing tgε26^+/+^ genomic clones that also contained hCD3ε sequence (left). Physical map of the transgenic integration site at the *Sstr5* locus (middle). H: HindIII. i: probe for Southern blot analysis. TS1F, TS1R, and CD3e9130F: primers for genomic PCR analysis. Genomic PCR analysis using TS1F/TS1R primers (lanes 1 and 2) and CDe9130F/TS1R primers (lanes 3 and 4) (right). (F, G) Southern blot analysis of WT and tgε26^+/+^ genomic DNA digested with the indicated restriction enzymes using the indicated probes. (H) Physical map of the transgenic integration site at the *Metrn* locus (left). N: NcoI. q: probe for Southern blot analysis. m7900, m8100, and CD3e23010F: primers for genomic PCR analysis. i-1R, i-1F, i-2R, and i-2F: primers for inverse and nested PCR amplification. Sequence of tgε26^+/+^ genomic clones containing both *Metrn* and hCD3ε sequences (middle). Genomic PCR analysis using m7900/m8100 primers (lanes 1 and 2) and m7900/CD3e23010F primers (lanes 3 and 4) (right). (I, J) PFGE Southern blot analysis of WT and tgε26^+/+^ genomic DNA digested with the indicated restriction enzymes using the indicated probes. (K) Quantitative genomic PCR analysis of WT (open bars) and tgε26^+/+^ (black bars) genomic DNA. Primers 1–7 are shown in (A). ct-1 and ct-2 are the genomic regions from chromosome 6 used as controls. Primer sequences are shown in Supplementary Table S3. Data were normalized to ct-1 signals. Means and standard errors of 3 independent measurements are shown. (L) Configuration of the tgε26 allele.

We next performed Southern blot analysis of this genomic region using pulse field gel electrophoresis (PFGE) of restriction enzyme-digested genomic DNA fragments. We found that among probes complementary to the 1 Mb genomic region between D17mit26-mit80-1 and D17mit 26-mit80-24 (Fig. 2A, probes a–g), a probe specific for the *Sstr5* coding sequence (probe c) detected a unique SalI fragment of approximately 200 kb in genomic DNA isolated from tgε26^+/+^ but not wild-type (WT) mice (Fig. 2B). The 200 kb SalI fragment was also detected by an hCD3ε probe, suggesting that this fragment contained the hCD3ε transgene (Fig. 2C). Interestingly, an 84 kb SalI fragment detected in the WT genome did not disappear in the genome of tgε26^+/+^ homozygous mice (Fig. 2B). Moreover, a probe specific for *Tekt4* coding sequence (Fig. 2A, probe b), which is located only 19 kb away from the *Sstr5* probe and which detected the same 84 kb SalI fragment as the *Sstr5* probe, failed to detect the 200 kb SalI fragment in tgε26^+/+^ genomic DNA (Fig. 2D). The 200 kb SalI fragment unique to tgε26^+/+^ genomic DNA was also not detectable by any other probes from this region (Fig. 2A, probes a, d, e, f, g) (data not shown). These results indicate that the hCD3ε transgene in the tgε26 allele is inserted at the *Sstr5* locus while preserving the neighboring *Tekt4* sequence.

To identify the integration site, a genomic library was constructed for tgε26^+/+^ mice and screened using the *Sstr5* probe. Sequence analysis of *Sstr5* ^+^ clones revealed that two of the clones contained a sequence identical to that beginning at nt 9497 of the 24 kb hCD3ε transgene (in intron 2 of the hCD3ε genomic sequence) but that this sequence was connected to the *Sstr5* gene 14401 bp 3′ of the transcription initiation site (Fig. 2E). PCR analysis showed that genomic DNA from tgε26^+/+^ but not WT mice was amplified by primers specific for hCD3ε transgenic sequence (CD3e9130F) and *Sstr5* genomic sequence (TS1R) (Fig. 2E, lanes 3, 4). However, PCR primers designed to amplify the sequence between 14271 bp and 14532 bp, 3′ of the *Sstr5* transcription initiation site (TS1F and TS1R), where the hCD3ε transgene is inserted, successfully amplified genomic DNA from tgε26^+/+^ as well as from WT mice (Fig. 2E, lanes 1 and 2). Southern blot analysis verified that a probe specific for *Sstr5* genomic sequence (Fig. 2A, E, probe i) detected a unique HindIII fragment of 4.1 kb in genomic DNA isolated from tgε26^+/+^ but not WT mice (Fig. 2F). Again, the 2.9 kb HindIII fragment detected in the WT genome was also present in the genome of tgε26^+/+^ mice (Fig. 2F). These results indicate that the hCD3ε transgene is inserted in the *Sstr5* locus of the tgε26 allele, but that the sequence between the *Sstr5* and *Tekt4* loci is preserved in tgε26^+/+^ mice.

It should be noted that the 200 kb tgε26-specific SalI fragment reproducibly showed greater signal intensity than the co-detected 84 kb fragment found in both WT and tgε26 genomic DNA (Fig. 2B), whereas the 4.1 kb tgε26-specific HindIII fragment was comparable in intensity to the co-detected 2.9 kb fragment found in WT and tgε26 genomic DNA (Fig. 2F). The *Sstr5* probe used in Fig. 2B (probe c) detected a comparable amount of the tgε26-specific fragment, and the fragment shared by WT and tgε26 genomic DNA after genomic DNA was digested with EcoRI (data not shown). Thus, the differential intensity of the 200 kb and 84 kb fragments (Fig. 2B) may reflect a difference in methylation status, which is known to affect SalI digestion.

The finding of a transgenic insertion at the *Sstr5* locus with preservation of the neighboring sequence suggested that this genomic region is duplicated in the tgε26 allele. To better understand transgene integration in the tgε26 allele, we performed additional Southern blot analysis using various probes complementary to the candidate genomic region (Fig. 2A, probes h to q). We found that a probe specific for sequence 7 kb 5′ of the *Metrn* gene (Fig. 2A, probe q), approximately 300 kb away from the *Sstr5* locus, detected a unique NcoI fragment of 3.3 kb in genomic DNA isolated from tgε26^+/+^ but not WT mice while retaining the 4.2 kb WT fragment of tgε26^+/+^ genomic DNA (Fig. 2G). To sequence the transgenic insertion at the *Metrn* locus, genomic DNA from tgε26^+/+^ mice was digested with NcoI, self-ligated, and amplified using inversely oriented PCR primers (Fig. 2H, i-1F, i-1R, i-2F, and i-2R). Amplified PCR products were cloned and sequenced. We found several clones containing hCD3ε sequence with nt 290 of the transgene connected to sequence 7689 bp 5′ of the *Metrn* gene (Fig. 2H). Genomic PCR analysis confirmed that a primer specific for the hCD3ε transgene (CD3e23010F) and a primer specific for the genomic sequence 5′ of the *Metrn* locus (m7900) amplified tgε26^+/+^, but not WT, genomic DNA (Fig. 2H, lanes 3 and 4). Primers specific to the genomic sequences interrupted by the transgenic insertion (m7900 and m8100) amplified both tgε26^+/+^ and WT genomic DNA (Fig. 2H, lanes 1 and 2). In PFGE Southern blot analysis, no other transgene integration site was detected in the candidate genomic region (Fig. 2I, J, and data not shown). These results indicate that in the tgε26 allele, in addition to the insertion at *Sstr5* locus, the hCD3ε transgene is inserted between the *Metrn* and *Fbx116* loci without disrupting neighboring sequences.

### Tgε26 transgenic insertion is accompanied by a genomic duplication spanning 323 kb

The above results revealed that in the tgε26 allele, multiple copies of the hCD3ε transgene spanning more than 200 kb are found in the region between the *Sstr5* and *Metrn* loci of chromosome 17, but that these insertions do not disrupt the intervening sequence. Therefore we speculated that transgenic insertion in the tgε26 allele is accompanied by duplication of the neighboring genomic region, as previously demonstrated in other transgenic mice [10]–[13]. Quantitative PCR analysis of genomic DNA isolated from WT and tgε26^+/+^ mice showed that the amount of DNA in the genomic region between the *Sstr5* and *Metrn* loci (Fig. 2A, primer sets 2 to 6) was approximately 2-fold higher in the tgε26^+/+^ genome than in the WT genome (Fig. 2K). The amount of DNA in the neighboring sequences of the *Tekt4* and *Fbxl16* loci (Fig. 2A, primer sets 1 and 7) and in two additional control sequences located on chromosome 6 (Fig. 2K, ct-1 and ct-2) was not increased in the tgε26^+/+^ genome (Fig. 2K). The undistorted amplification curves for these quantitative PCR reactions indicate that the 2-fold elevation of DNA signal amplified by primer sets 2–6 reflects the presence of 2-fold greater genomic DNA before amplification rather than aberrant amplification efficiency in certain PCR reactions (Supplementary Fig. S1). These results indicate that transgenic integration of the tgε26 allele is accompanied by duplication of a 323 kb genomic region between the *Sstr5* and *Metrn* loci (Fig. 2L).

### Genes surrounding the integrated tgε26 transgene are not disrupted

**Figure 3:**
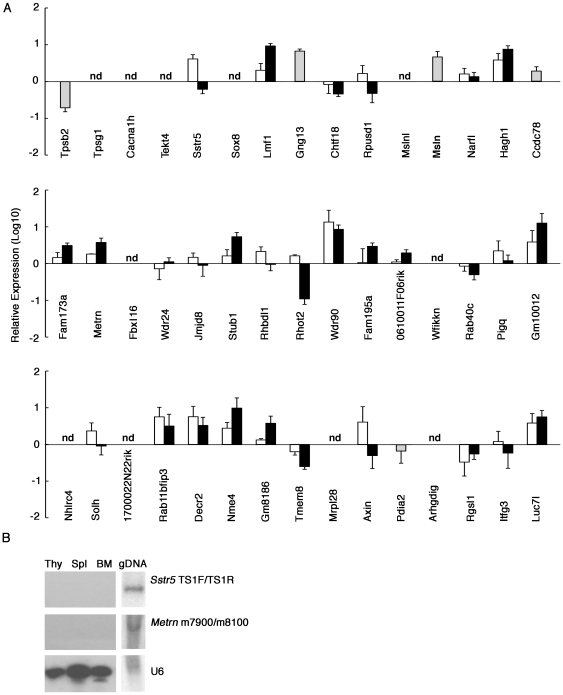
Expression of genes surrounding the hCD3ε transgenic integration site in tgε26^+/+^ thymocytes. (A) Quantitative PCR analysis of mRNA expression in neonatal tgε26^+/−^ (open bars) and tgε26^+/+^ (black bars) thymocytes. Results were normalized to GAPDH mRNA and quantitated relative to DN1 cells isolated from WT mice. The expression of *Tpsb2*, *Gng13*, *Msln*, *Ccdc78* and *Pdia2* in tgε26^+/+^ thymocytes (gray bars) was quantitated relative to tgε26^+/−^ thymocytes, because expression of these genes was not detected in WT thymocytes. Means and standard errors of 3 independent measurements are shown. nd: not detected. Primer sequences are shown in Supplementary Table S4. (B) RNA isolated from thymocytes (Thy), splenocytes (Spl), bone marrow cells (BM), and genomic DNA (gDNA) of WT mice was electrophoresed on a polyacrylamide gel and then blotted and probed for TS1F/TS1R sequence (Fig. 2E), m7900/m8100 sequence (Fig. 2H) and U6 small nuclear RNA. Representative results from two independent experiments are shown.

The genomic duplication of the region between the *Sstr5* and *Metrn* loci in the tgε26 allele implies that none of the sequences surrounding the transgenic insertion should be lost in the tgε26 allele. Indeed, of the 45 genes located within the functionally mapped genomic region between D17mit26-mit80-1 and D17mit 26-mit80-24 (Fig. 1B), none showed severely abrogated expression in tgε26^+/+^ newborn thymocytes, which remained B220^−^CD4^−^CD8^−^ before the development of B220^+^ B cells [3], as compared to newborn B220^−^CD4^−^CD8^−^ thymocytes isolated from TCRβ^−/−^δ^−/−^ mice (Supplementary Fig. S2). However, the expression of *Tpsb2*, *Gng13*, *Msln*, *Stub1*, and *Pdia2* in this genomic region was more than 10-fold increased in tgε26^+/+^ thymocytes (Supplementary Fig. S2), which may reflect enhanced expression of these genes due to transcriptional accessibility of the neighboring hCD3ε transgene in immature T-lymphoid cells. We also compared the expression of these 45 genes in DN1 thymocytes isolated from tgε26^+/+^, tgε26^+/−^, and WT newborn mice, and found that none were severely abrogated specifically in tgε26^+/+^ thymocytes (Fig. 3A). The undistorted amplification curves for these quantitative RT-PCR reactions indicate that the PCR signals measured in these experiments faithfully reflect the amount of mRNA transcripts expressed in the cells (Supplementary Fig. S3).

Interestingly, expression of *Rhot2* was approximately 10-fold reduced in tgε26^+/+^ thymocytes compared to WT and tgε26^+/−^ thymocytes (Fig. 3A). However, we found using OP9-DL1 co-culture [14] that over-expression of *Rhot2* did not rescue defective T cell development from tgε26^+/+^ lymphoid progenitor cells (data not shown), suggesting that the reduced expression of *Rhot2* is not primarily responsible for defective T cell development in tgε26^+/+^ mice. Expression of *Lmf1*, *Hagh1*, *Wdr90*, *Gm10012* and *Nme4* was approximately 10-fold elevated in tgε26^+/+^ and tgε26^+/−^ thymocytes compared to WT thymocytes (Fig. 3A). In addition, expression of *Tpsb2*, *Gng13*, *Msln*, *Ccdc78* and *Pdia2* was detected in tgε26^+/+^ and tgε26^+/−^ thymocytes but not in WT thymocytes (Fig. 3A, gray bars), and expression of *Gng13* and *Msln* was higher in tgε26^+/+^ thymocytes than in tgε26^+/−^ thymocytes (Fig. 3A). However, we found that over-expression of *Gng13* or *Msln* did not perturb T cell development from WT lymphoid progenitor cells in OP9-DL1 co-culture (data not shown), suggesting that increased expression of *Gng13* or *Msln* is not primarily responsible for defective T cell development in tgε26^+/+^ mice. It was previously reported that over-expression of *Axin*, a gene localized to this genomic region, impairs T cell development [15]. However, the expression of *Axin* was not greatly altered in tgε26^+/+^ thymocytes.

Nonetheless, among the 45 genes examined, we detected no mutations in the coding sequences of 42 genes, while the other 3 genes, *Tpsg1*, *Mslnl* and *Mrpl28*, have extremely low levels of expression in lymphoid and other tissues (data not shown). Thus, none of the genes surrounding the transgenic insertion site are completely lost in the tgε26 allele. These results suggest that the severe T cell deficiency seen in tgε26 mice is not due to disruption of the genes surrounding the transgenic integration site. However, it is possible that gross alterations of gene expression profiles in this genomic region may contribute to the defective T cell development of tgε26 mice.

It is known that non-coding RNAs are involved in the regulation of many genes [16], [17]. We finally examined whether transgenic insertion disrupts neighboring non-coding RNAs in tgε26^+/+^ mice. No non-coding RNAs have been identified within the genomic sequence 58 kb from the *Metrn* transgenic integration site and 216 kb from the *Sstr5* transgenic integration site (Ensembl and miRBase databases). Moreover, probes complementary to *Sstr5* and *Metrn* sequences (the TS1F/TS1R and m7900/m8100 PCR products shown in Fig. 2E and 2H) gave no signals when hybridized using blots with total RNAs isolated from thymocytes, splenocytes, and bone marrow cells from WT mice, whereas U6 small nuclear RNA was detected in these cells using this method (Fig. 3B). These results indicate that non-coding RNAs are not located in the transgenic integration site and are not disrupted in the tgε26 allele.

### Conclusion

We have identified the transgenic integration site of the tgε26^+/+^ allele and shown that tgε26 transgenic insertion is accompanied by duplication of neighboring sequences and that no neighboring genes are lost in the tgε26 allele. These results indicate that the severe T cell deficiency unique to homozygous tgε26 mice is not due to gene disruption by transgenic integration. It is possible that transgenic insertion alters the function of *cis*-acting elements located in the neighboring sequences that regulate remotely encoded genes that are critical for early T cell development. It is also possible that T cell deficiency in tgε26 mice is independent of genomic alterations, and is completely due to the aberrantly expressed hCD3ε chain in homozygous tgε26 lymphoid progenitor cells. Further elucidation of the mechanisms underlying T cell deficiency in tgε26 mice may lead to a better understanding of the molecular mechanisms of early T cell development.

## Methods

### Ethics Statement

This study utilized experiments using mice. All experiments using mice were performed with consent from the Animal Experimentation Committee of the University of Tokushima (Toku Dobutsu 08059). The study did not involve human experiments.

### Mice

Tgε26 human CD3ε-transgenic mice [1] and TCR-β/TCR-δ double knockout mice [18], [19] were described previously. C57BL/6 mice were purchased from SLC Japan. All experiments using mice were performed with consent from the Animal Experimentation Committee of the University of Tokushima.

### Linkage analysis

Homozygous tgε26 mice were crossed with tgε26×C57BL/6 heterozygous mice. Linkage analysis was performed as described previously [20], [21]. To determine phenotypes, peripheral blood white cells were stained with FITC-conjugated antibody for TCRβ and biotinylated antibody for CD3 followed by staining with phycoerythrin-conjugated streptavidin. Two-color flow cytometry analysis was performed using FACS-Calibur (BD Biosciences).

### Southern blot analysis

For PFGE analysis, genomic DNA was embedded in agarose using the CHEF Genomic DNA Plug Kit (Bio-Rad), digested with the indicated restriction enzymes, and electrophoresed in 1% agarose using the CHEF Mapper XA system (Bio-Rad). For conventional analysis, genomic DNA was digested and electrophoresed in 1% agarose. DNA was transferred to a nylon membrane (Hybond-N+, GE Healthcare). Probes were labeled with [α-^32^P] dCTP using the Random Primer DNA Labeling Kit (Takara Bio). The membranes were pre-hybridized for 30 min and hybridized overnight at 42^o^C in 50% formamide, 0.25 M NaCl, 0.12 M Na_2_HPO_4_, 7% SDS, and 100 µg/ml denatured herring sperm DNA. Hybridized membranes were washed in 2× SSC, 0.1%SDS at 60^o^C for 30 min and then in 0.2× SSC, 0.1%SDS at 60^o^C for 30 min, and exposed to Kodak Biomax MS X-ray film at −80^o^C with an intensifying screen.

### Molecular cloning

For phage library construction, tgε26^+/+^ genomic DNA was partially digested with Sau3AI and cloned into the BamHI site of the bacteriophage λEMBL3 (Promega). Recombinant bacteriophages were screened with ^32^P-labeled *Sstr5* probe. For inverse PCR, tgε26^+/+^ genomic DNA was digested with NcoI, and 2–3 kb DNA fragments were extracted from agarose gel using the QIAquick Gel Extraction Kit (Qiagen) and were self-ligated using T4 DNA ligase (Takara) and PCR amplified using the i-1F (5′-TATCCGAGCCAAATGTGCCA-3′) and i-1R (5′-AGATAGAAACCTCAATGCCCA-3′) primers. Primers used for nested PCR were i-2F (5′-GATGTTCCAAAAGCGTCATCAGG-3′) and i-2R (5′-GCTGAAGTGCCAGGC TAATG-3′). Amplified fragments were cloned into the pCRII-X vector. DNA was sequenced using a 3100 Genetic Analyzer (Applied Biosystems).

### Genomic PCR analysis

Genomic DNA was PCR-amplified using the following primers: TS1F (5′-AGTTGCCCATGCTTCAGCGAGTA-3′), TS1R (5′-CTGCCCATCCCAGTACATGCCT -3′), CD3e9130F (5′-GAGCGAAGCTCCACTCCTTGTT-3′), m7900 (5′-AGAGCC CAAGGAACAAGGGT-3′), m8100 (5′-CAACCACATGGTGGCTCACA-3′) and CD3e23010F (5′-GTGGTTGGGCCACACTTTCA-3′). For quantitative PCR analysis, genomic DNA was PCR-amplified and analyzed using SYBR Green PCR Master Mix (Takara) and a 7900HT Sequence Detection System (Applied Biosystems). Amplified signals were confirmed to be single bands using gel electrophoresis.

### Cell sorting

Thymocytes were labeled with biotinylated CD4 and CD8 antibodies. CD4^−^CD8^−^ DN cells were purified by depleting CD4^+^ cells and CD8^+^ cells using a magnetic cell sorter (Miltenyi Biotec). DN cells were stained with FITC-conjugated antibody specific for CD44 and phycoerythrin-conjugated antibody specific for CD25 (BioLegend). CD44^+^CD25^−^ DN1 cells were sorted by FACS AriaII (BD Biosciences).

### Quantitative RT-PCR analysis

Total RNA isolated from neonatal thymocytes was treated with RNase-free DNaseI (Takara) and reverse-transcribed using SuperScriptIII reverse transcriptase (Invitrogen). cDNA was PCR-amplified and analyzed using SYBR Green PCR Master Mix (Takara) and a 7900HT Sequence Detection System. Amplified signals were confirmed to be single bands using gel electrophoresis, and were normalized to GAPDH levels.

### RNA blot analysis

Total RNA (10 µg) was loaded onto a 15% polyacrylamide gel containing 7M urea, transferred to a nylon membrane, and hybridized with genomic DNA fragments amplified from C57BL/6 mice using TS1F/TS1R or m7900/m8100 primers. A U6 RNA probe was also used. Hybridization was carried out at 42^o^C overnight as described for Southern blot analysis.

## Supporting Information

Figure S1 Amplification curves for quantitative genomic PCR reactions. PCR primers (ct-1, ct-2, and 1–7) are indicated at the top of each plot.
(0.68 MB TIF)

Figure S2 Quantitative PCR analysis of mRNA expression of genes surrounding the hCD3ε transgenic integration site in neonatal tgε26+/+ thymocytes. Results were normalized to GAPDH mRNA and quantitated relative to TCRβ−/−δ−/− neonatal thymocytes. Means and standard errors of 3 independent measurements are shown. nd: not detected.
(0.15 MB TIF)

Figure S3 Amplification curves for the quantitative RT-PCR reactions. Names of the genes represented are indicated at the top of each plot.
(0.86 MB TIF)

Table S1 (0.37 MB TIF)

Table S2 (0.21 MB TIF)

Table S3 (0.20 MB TIF)

Table S4 (0.94 MB TIF)

## References

[1] Wang B, Biron C, She J, Higgins K, Sunshine MJ (1994). A block in both early T lymphocyte and natural killer cell development in transgenic mice with high-copy numbers of the human CD3E gene.. Proc Natl Acad Sci USA.

[2] Wang B, Hollander GA, Nichogiannopoulou A, Simpson SJ, Orange JS (1996). Natural killer cell development is blocked in the context of aberrant T lymphocyte ontogeny.. Int Immunol.

[3] Tokoro Y, Sugawara T, Yaginuma H, Nakauchi H, Terhorst C (1998). A mouse carrying genetic defect in the choice between T and B lymphocytes.. J Immunol.

[4] Wang B, Simpson SJ, Holländer GA, Terhorst C (1997). Development and function of T lymphocytes and natural killer cells after bone marrow transplantation of severely immunodeficient mice.. Immunol Rev.

[5] Holländer GA, Wang B, Nichogiannopoulou A, Platenburg PP, van Ewijk W (1995). Developmental control point in induction of thymic cortex regulated by a subpopulation of prothymocytes.. Nature.

[6] Levelt CN, Wang B, Ehrfeld A, Terhorst C, Eichmann K (1995). Regulation of T cell receptor (TCR)-beta locus allelic exclusion and initiation of TCR-alpha locus rearrangement in immature thymocytes by signaling through the CD3 complex.. Eur J Immunol.

[7] Guimond MJ, Wang B, Fujita J, Terhorst C, Croy BA (1996). Pregnancy-associated uterine granulated metrial gland cells in mutant and transgenic mice.. Am J Reprod Immunol.

[8] van Ewijk W, Holländer G, Terhorst C, Wang B (2000). Stepwise development of thymic microenvironments in vivo is regulated by thymocyte subsets.. Development.

[9] Roberts NA, Desanti GE, Withers DR, Scott HR, Jenkinson WE (2009). Absence of thymus crosstalk in the fetus does not preclude hematopoietic induction of a functional thymus in the adult.. Eur J Immunol.

[10] Smiraglia DJ, Wu C, Ellsworth MK, Ratty AK, Chapman VM (1997). Genetic characterization of the chromosomal rearrangements that accompany the transgene insertion in the chakragati mouse mutant.. Genomics.

[11] Wen XY, Bryce DM, Breitman ML (1998). Characterization of lpd (lipid defect): a novel mutation on mouse chromosome 16 associated with a defect in triglyceride metabolism.. Hum Mol Genet.

[12] Morgan D, Turnpenny L, Goodship J, Dai W, Majumder K (1998). Inversin, a novel gene in the vertebrate left-right axis pathway, is partially deleted in the inv mouse.. Nat Genet.

[13] Vermeire L, Ibrahimi A, Voet T, Umans L, Coddens K (2009). Essential validation of gene trap mouse ES cell lines: a test case with the gene Ttrap.. Int J Dev Biol.

[14] Schmitt MT, Zuniga-Pflücker JC (2002). Induction of T cell development from hematopoietic progenitor cells by Delta-like-1 in vitro.. Immunity.

[15] Hsu W, Shakya R, Costantini F (2001). Impaired mammary gland and lymphoid development caused by inducible expression of Axin in transgenic mice.. J Cell Biol.

[16] Mattick JS, Makunin IV (2006). Non-coding RNA.. Hum Mol Genet.

[17] Goodrich JA, Kugel JF (2006). Non-coding-RNA regulators of RNA polymerase II transcription.. Nat Rev Mol Cell Biol.

[18] Mombaerts P, Clarke AR, Rudnicki MA, Iacomini J, Itohara S (1992). Mutations in T-cell antigen receptor genes α and β block thymocyte development at different stages.. Nature.

[19] Itohara S, Mombaerts P, Lafaille J, Iacomini J, Nelson A (1993). T cell receptor δ gene mutant mice: independent generation of αβ T cell and programmed rearrangement of γδ TCR genes.. Cell.

[20] Kanemoto N, Hishigaki H, Miyakita A, Oga K, Okuno S (1998). Genetic dissection of “OLETF”, a rat model for non-insulin-dependent diabetes mellitus.. Mamm Genome.

[21] Watanabe TK, Ono T, Mizoguchi-Miyakita A, Yamasaki Y, Kanemoto N (2000). Characterization of newly developed SSLP markers for the rat.. Mamm Genome.

