# Supplementary figures and images for: Identification of the Transgenic Integration Site in Immunodeficient tgε26 Human CD3ε Transgenic Mice
Source: PLoS One. 2010 Dec 22;5(12):e14391. doi: 10.1371/journal.pone.0014391 (PMC3008721; doi:10.1371/journal.pone.0014391)

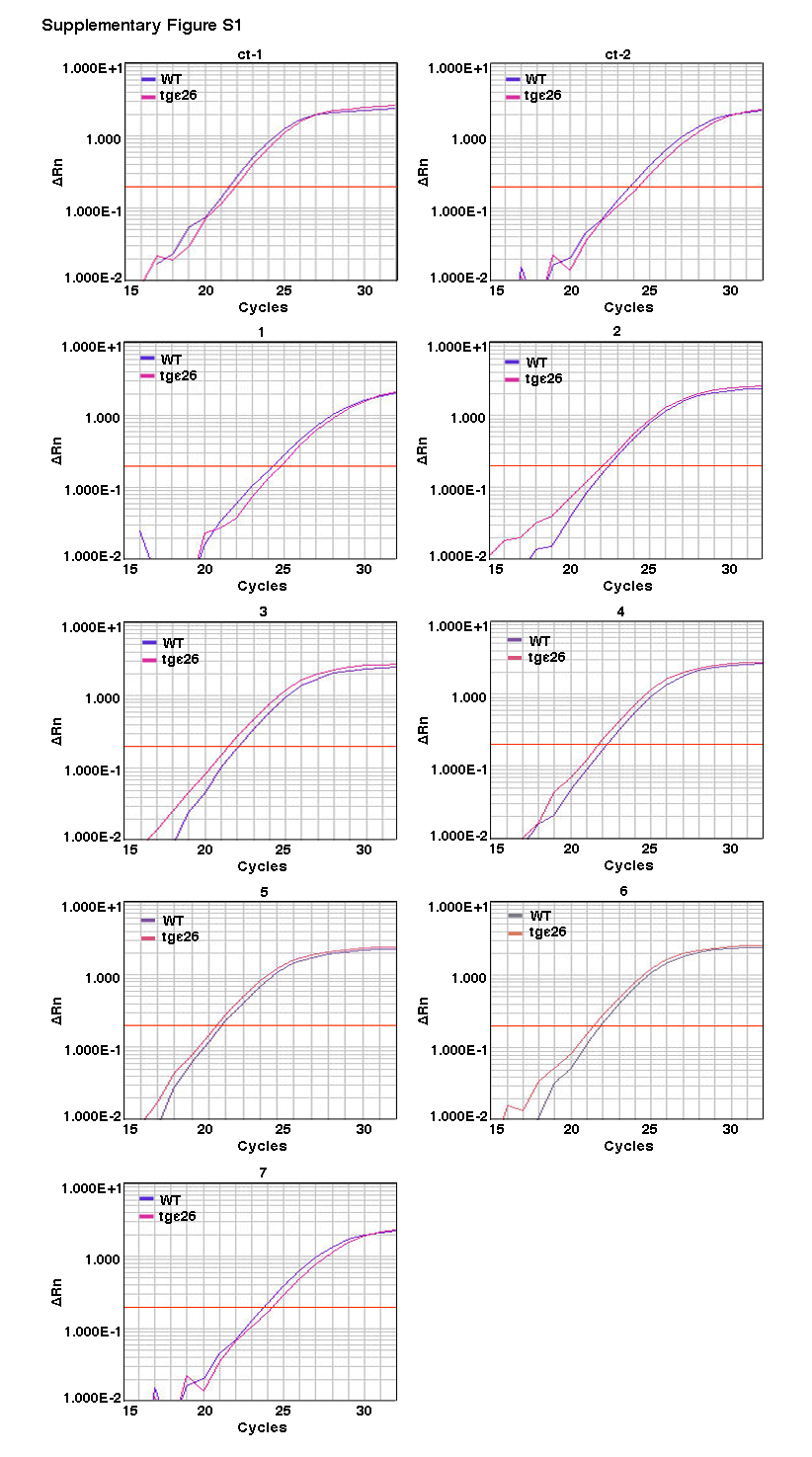

Supplement: Figure S1 — Amplification curves for quantitative genomic PCR reactions. PCR primers (ct-1, ct-2, and 1–7) are indicated at the top of each plot. (0.68 MB TIF) [file pone.0014391.s001.tif]

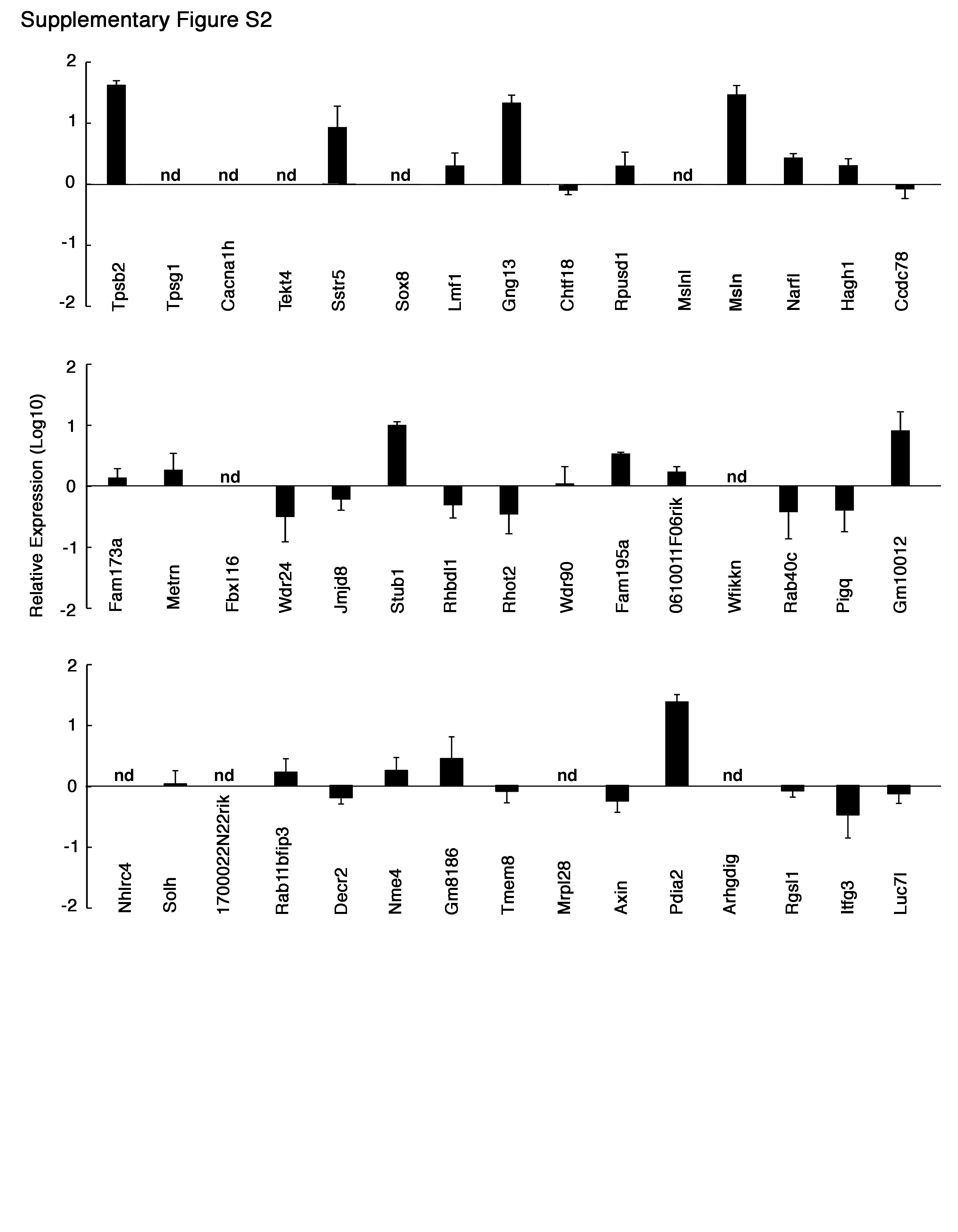

Supplement: Figure S2 — Quantitative PCR analysis of mRNA expression of genes surrounding the hCD3ε transgenic integration site in neonatal tgε26+/+ thymocytes. Results were normalized to GAPDH mRNA and quantitated relative to TCRβ−/−δ−/− neonatal thymocytes. Means and standard errors of 3 independent measurements are shown. nd: not detected. (0.15 MB TIF) [file pone.0014391.s002.tif]

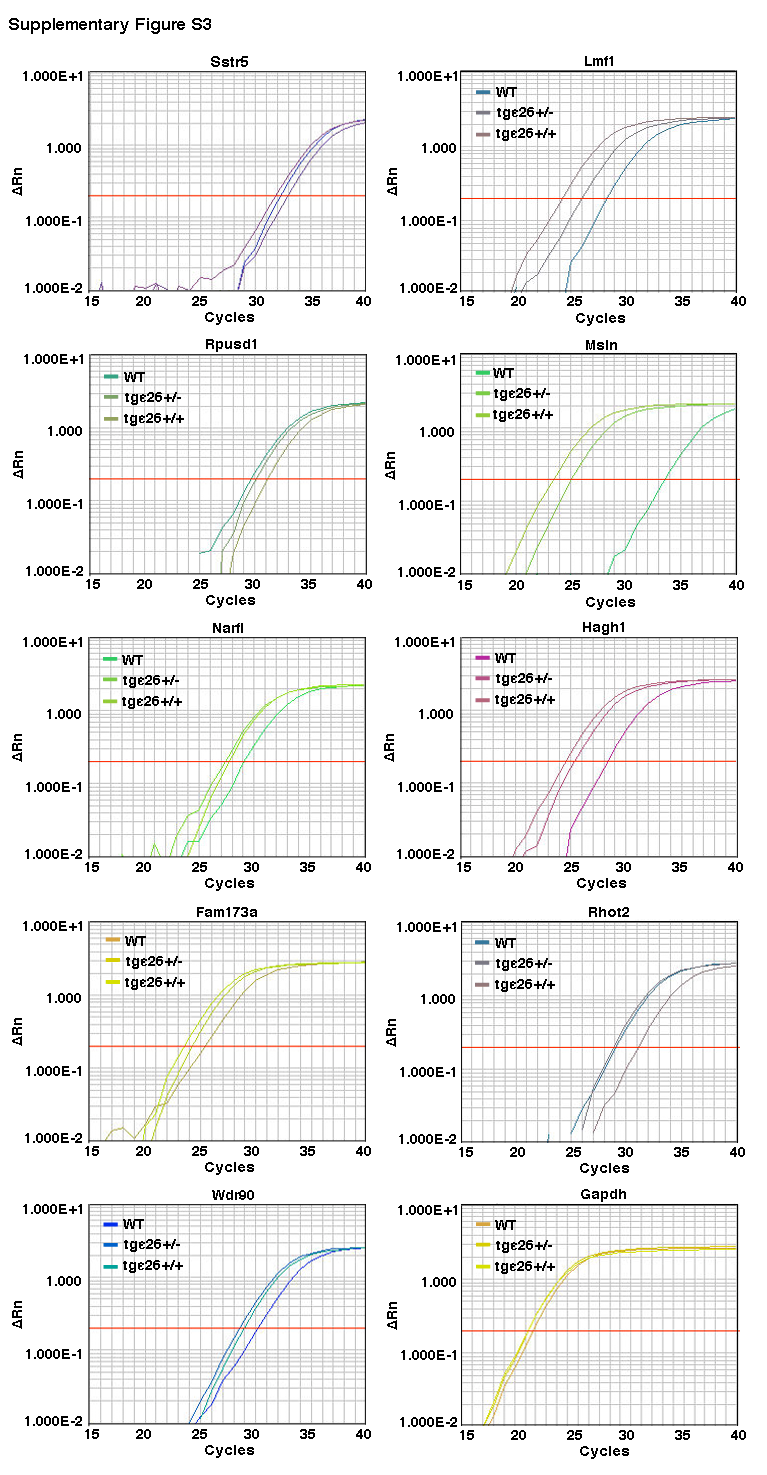

Supplement: Figure S3 — Amplification curves for the quantitative RT-PCR reactions. Names of the genes represented are indicated at the top of each plot. (0.86 MB TIF) [file pone.0014391.s003.tif]

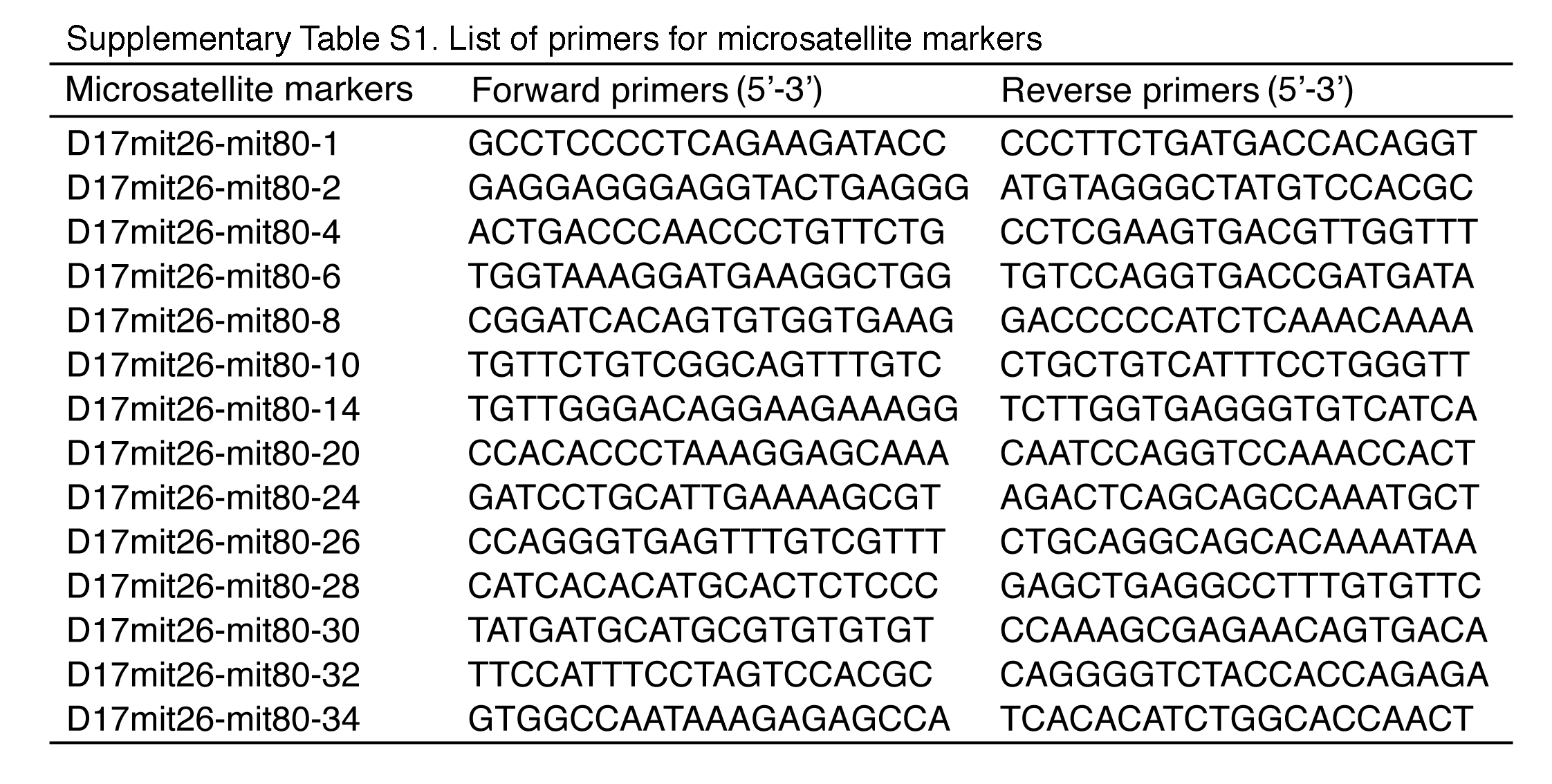

Supplement: Table S1 — (0.37 MB TIF) [file pone.0014391.s004.tif]

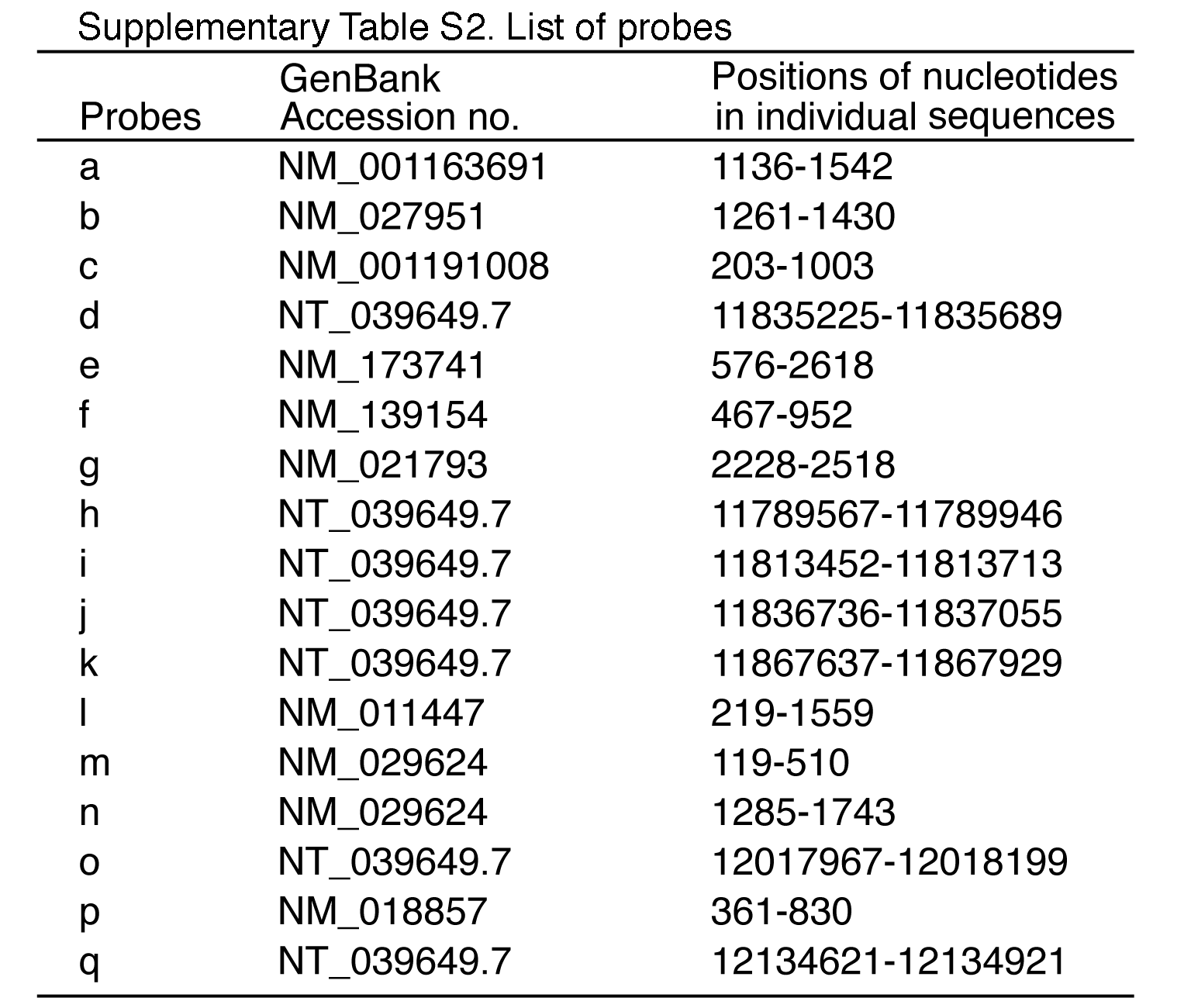

Supplement: Table S2 — (0.21 MB TIF) [file pone.0014391.s005.tif]

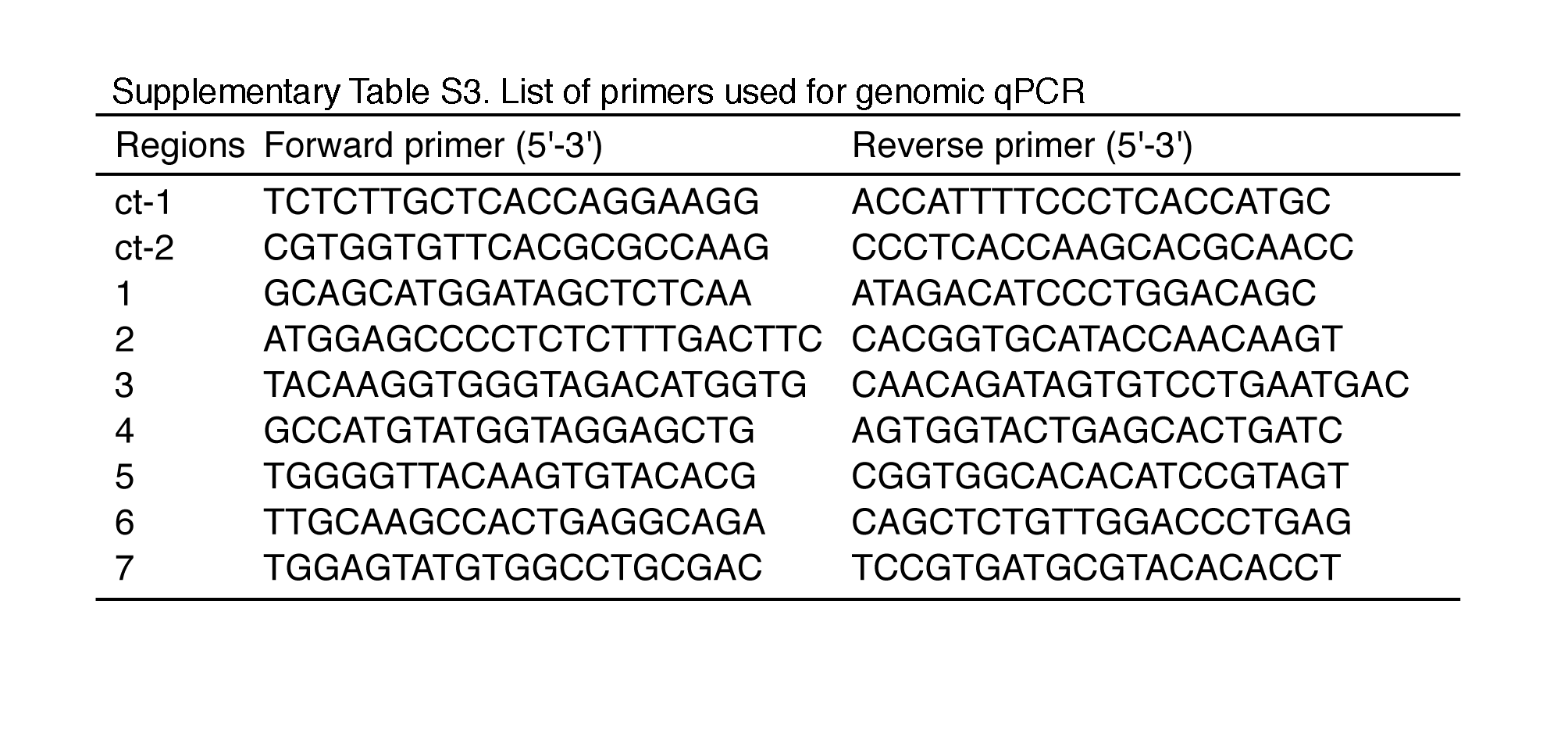

Supplement: Table S3 — (0.20 MB TIF) [file pone.0014391.s006.tif]

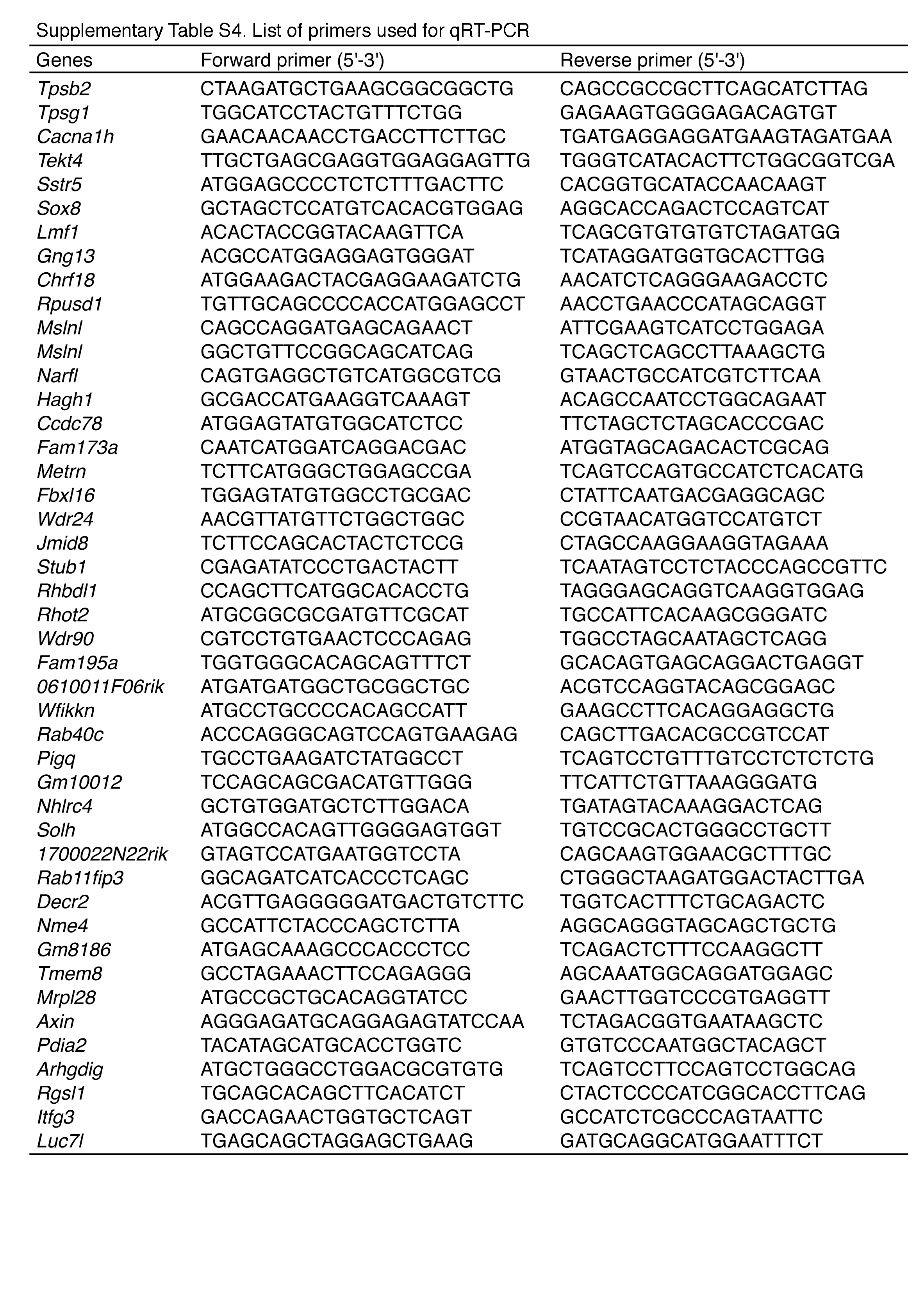

Supplement: Table S4 — (0.94 MB TIF) [file pone.0014391.s007.tif]
